# An Unusual Pediatric Case of Allen Key Penetrating Trauma in Maxillofacial Region

**DOI:** 10.1155/2018/5086154

**Published:** 2018-04-23

**Authors:** Feride Fatma Görgülü, Orhan Görgülü, Ayşe Selcan Koç, Fatma Yasemin Öksüzler

**Affiliations:** ^1^Radiology Department, University of Health Sciences Adana City Research and Training Hospital, Adana, Turkey; ^2^ENT Department, University of Health Sciences Adana City Research and Training Hospital, Adana, Turkey

## Abstract

Paranasal sinus (PNS) foreign bodies are not common. They are usually due to penetrating trauma and iatrogenic events. On imaging, radiopaque foreign bodies can easily be detected by X-ray views of PNS. CT scan may be necessary to evaluate the exact location of foreign body in some cases. Foreign body in the PNS should be removed as early as possible. Approach and technique of its removal depend upon its size, shape, and location. Nasal endoscopic examination can be helpful for these cases. We present a pediatric girl case of penetrating FB injury inserted into the maxillary sinus towards nasopharynx in a suddenly braking car.

## 1. Introduction

Paranasal sinus foreign bodies (PNS FBs) are commonly secondary to gunshots and accidental sharp materials [1–3]. There was a reported case of an arrow, wooden foreign body, and wristwatch in the PNS. Foreign bodies in the PNS should be removed as early as possible. A retained foreign body can lead to sinusitis, cutaneous fistula, and foreign body granuloma formation [4–6].

Advanced imaging methods may be necessary in some cases whereas simpler radiological method can give us enough anatomical information about the FB in some other cases. We present a pediatric girl case of penetrating FB injury inserted into the maxillary sinus towards nasopharynx in a suddenly braking car. We would like to draw attention to choosing the radiological algorithm as first simpler to advanced ones in the examination of such patients besides being able to prevent cases like this trauma inside car.

## 2. Case Report

**Figure 1:**
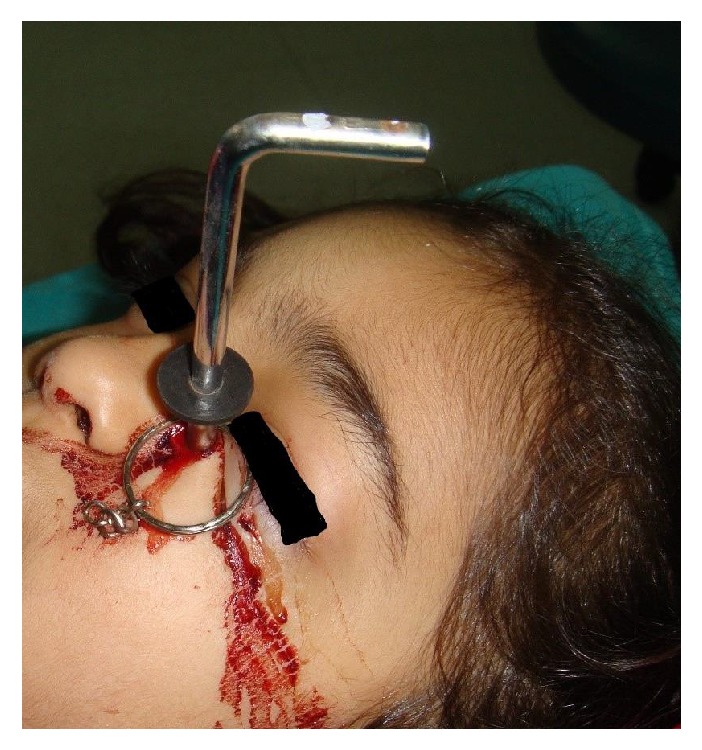
Photograph of the patient at the time of admission.

Nine-year-old pediatric girl patient came to the emergency department with the history of an Allen key injury on her left cheek (Figure 1). The family of the patient said that when the patient was traveling in the car, Allen key in her hand was inserted into her face because of the sudden brake.

**Figure 2:**
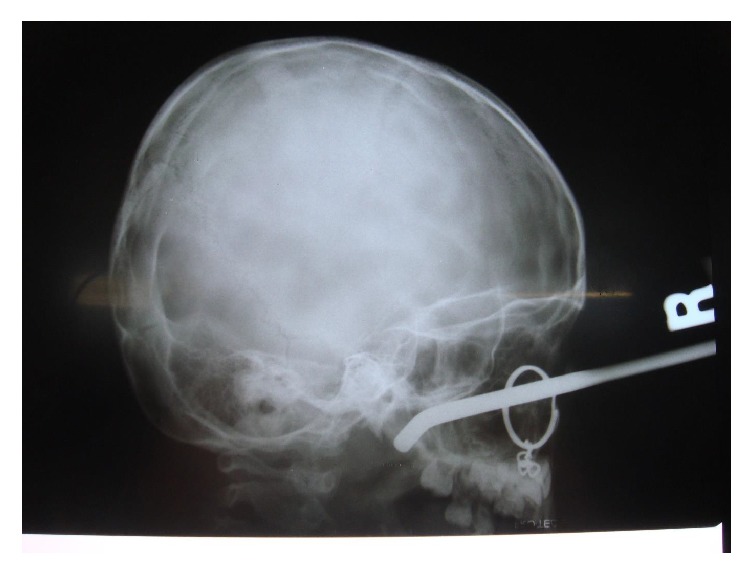
Preoperative X-ray of the skull lateral view, showing the Allen key in the left maxillary sinus to nasopharynx.

**Figure 3:**
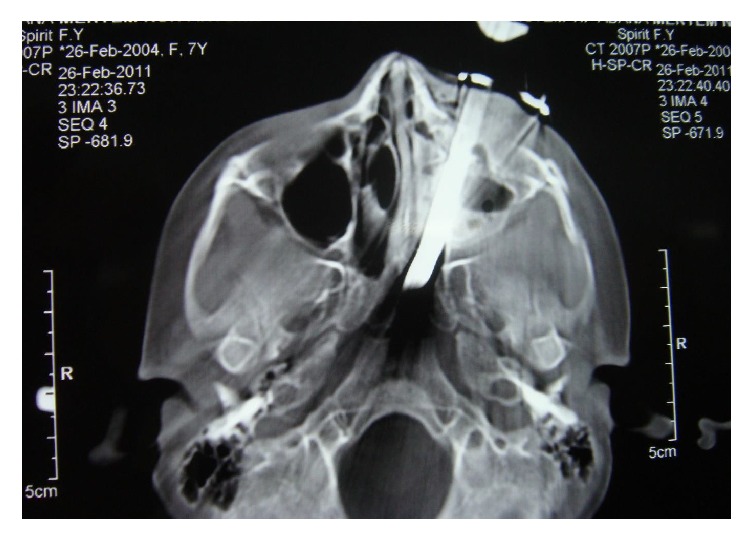
Foreign body visualised on CT at the time of admission.

On examination, an entry of foreign body was found on the upper left cheek. The tip of Allen key was seen at nasopharynx with diagnostic nasal endoscopy after the nasal decongestion. There were edema and tenderness over the left cheek. It revealed no hemorrhage of the turbinates or the nasal mucosa on anterior rhinoscopy. Firstly the lateral skull roentgenogram revealed Allen key in the left maxillary sinus to nasopharynx. The body of the Allen key has a very close relation to the floor of the orbit and the sphenoid sinus (Figure 2). This image was good enough for us, but the computed tomography (CT) was also used to make sure that the periorbital and skull base were damaged or not. CT of the maxillofacial region revealed no orbital and intracranial injury (Figure 3). The patient also had no neurological or vascular injury.

**Figure 4:**
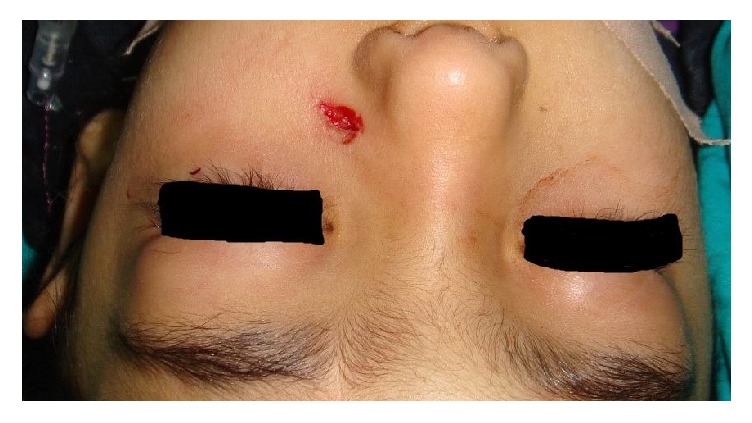
Photograph of the patient after removal.

The patient was immediately operated on under general anesthesia. During the operation, the foreign body was removed after short-term cauterization with monopolar cautery due to its metallic nature by making a millimetric incision on the adjacent skin tissue to prevent skin damage (Figure 4). This step was done to prevent active bleeding. The entry site and incision were sutured when no hemorrhage was seen after the foreign body was removed. The left nasal passages were again examined by nasal endoscopy and no abnormality was detected. Antibiotics were given for seven days to prevent possible infection. The patient was discharged on the same day of the operation.

At the 6-month follow-up, the patients' physical examination and radiological investigations were normal.

## 3. Discussion

Foreign bodies in the PNS are rare. It is difficult to predict the frequency of occurrence because of the small number of publications published in the literature. About 50% to 75% of all foreign bodies in the PNS are found in the maxillary sinuses [7–9]. Introduction of foreign bodies to the paranasal sinuses may occur through a variety of traumatic or iatrogenic events. Traumatic ones include pellets or bullets from gunshot injuries, wood, pieces of glass, and stones, while iatrogenic ones include teeth, dental cement, and pieces of broken forceps [10–12].

The diagnostic approach consists of a complete medical history, otolaryngological examination, and appropriate radiographs. Patients coming in the emergency with blast injuries and penetrating trauma should undergo radiological investigation before operation. Waters and lateral skull views are useful imaging tools in diagnosing FB and CT scan can be usefully used to evaluate the presence and exact location of FB [8, 13, 14].

Foreign bodies in the PNS should be removed as early as possible. A retained foreign body can lead to sinusitis, cutaneous fistula, foreign body granuloma formation, and even malignant mucosal alteration. The general treatment guidelines for a penetrating maxillofacial injury are to decompress, debride, and avoid neurovascular injury and the subsequent complications. A multidisciplinary surgical intervention is always required. There are a variety of techniques for removing paranasal sinus FBs. Location of foreign body may require a variation in operative technique. The surgical approach must be chosen according to the mechanisms, patterns, and materials of the injury. The classic open surgical techniques are preferred in large FBs, whereas small foreign bodies can easily be removed endoscopically [15–19].

The surgical technique used in this case was unusual because the tip and body of the metallic FB were inside the face. Therefore there was a possible risk of significant bleeding, if we prefer to remove it simply by pulling it from skin. We removed it after short-term cauterization with monopolar cautery by making a millimetric incision on the adjacent skin tissue to prevent skin damage.

This case is unusual and interesting for three reasons; first, the foreign body itself, a whole Allen key, is the first in the literature to our knowledge; second, it also draws attention to the causing form of trauma, a girl in vehicle having Allen key in her hand that was preventable cause; third, our aim is to emphasise choosing simpler radiological methods rather than complicated ones.

## 4. Conclusion

Foreign bodies in the paranasal sinuses vary in their size and location. Approach and technique of their removal depend upon their size, shape, and location. In some cases simpler radiological view can give us enough information; in the other ones advanced imaging like MRI and angiography may be necessary.
